# Unraveling Asymmetric Electrochemical Kinetics in Low-Mass-Loading LiNi_1_/_3_Mn_1_/_3_Co_1_/_3_O_2_ (NMC111) Li-Metal All-Solid-State Batteries

**DOI:** 10.3390/ma17205014

**Published:** 2024-10-14

**Authors:** Byoung-Nam Park

**Affiliations:** Department of Materials Science and Engineering, Hongik University, 72-1, Sangsu-dong, Mapo-gu, Seoul 04066, Republic of Korea; metalpbn@hongik.ac.kr

**Keywords:** low mass loading, solid electrolyte, Li-ion diffusivity, NMC111, electrode/electrolyte interface

## Abstract

In this study, we fabricated a Li-metal all-solid-state battery (ASSB) with a low mass loading of NMC111 cathode electrode, enabling a sensitive evaluation of interfacial electrochemical reactions and their impact on battery performance, using Li_1.3_Al_0.3_Ti_1.7_(PO_4_)_3_ (LATP) as the solid electrolyte. The electrochemical behavior of the battery was analyzed to understand how the solid electrolyte influences charge storage mechanisms and Li-ion transport at the electrolyte/electrode interface. Cyclic voltammetry (CV) measurements revealed the *b*-values of 0.76 and 0.58, indicating asymmetry in the charge storage process. A diffusion coefficient of 1.5 × 10^−9^ cm^2^⋅s^−1^ (oxidation) was significantly lower compared to Li-NMC111 batteries with liquid electrolytes, 1.6 × 10^−8^cm^2^⋅s^−1^ (oxidation), suggesting that the asymmetric charge storage mechanisms are closely linked to reduced ionic transport and increased interfacial resistance in the solid electrolyte. This reduced Li-ion diffusivity, along with the formation of space charge layers at the electrode/electrolyte interface, contributes to the observed asymmetry in charge and discharge processes and limits the rate capability of the solid-state battery, particularly at high charging rates, compared to its liquid electrolyte counterpart.

## 1. Introduction

Solid electrolytes offer enhanced safety compared to traditional liquid electrolytes, which are typically flammable and prone to leakage. In Li-metal batteries, using a solid electrolyte can significantly reduce the risk of thermal runaway and fire, making the battery safer for commercial and large-scale applications, such as electric vehicles and grid storage [1,2,3]. NMC111, a high-energy cathode material, benefits from the solid electrolyte’s ability to prevent dangerous side reactions, such as lithium dendrite growth, which can short-circuit the battery in liquid electrolyte systems [4].

Investigating the low mass loading of NMC111 in a Li-metal battery with a solid electrolyte allows researchers to focus on the electrode/electrolyte interface. This interface is crucial for determining battery performance, especially in solid-state systems where interfacial contact is more challenging compared to liquid electrolyte systems [5,6,7,8]. Studying low-mass NMC111 can reveal insights into how Li-ion transport and electrochemical reactions behave in a solid-state environment, potentially leading to improved interface stability and reduced resistance, which are key challenges for solid-state batteries.

From an industrial standpoint, high-mass-loading electrodes present numerous benefits, such as increased energy density and reduced manufacturing costs. However, these electrodes face critical challenges, including weak adhesion between the electrode materials and current collectors, leading to delamination and performance degradation. More importantly, limited electron/ion transport and restricted electron penetration depth have posed significant obstacles. To address these limitations, various strategies have been implemented to enhance electron/ion transport and improve performance. These approaches include optimizing conductive additives like carbon black, CNTs [9], and graphene [10] to boost electrical conductivity; modifying polymer binders to improve structural cohesion and transport efficiency [11]; and designing advanced 3D porous or nanostructured current collectors to facilitate more effective charge transfer [12,13,14]. Additionally, the development of nanostructured active materials with hierarchical porosity and low-tortuosity pathways has been shown to enhance ion diffusion and electron transport while maintaining material stability, thereby improving the overall electrochemical performance of high-mass-loading electrodes [13,15,16].

In contrast to the extensive efforts focused on optimizing high-mass-loading electrodes, the fundamental study of low-mass-loading electrodes has lagged behind [17]. Low mass loading provides a unique opportunity to closely study the electrode/electrolyte interface, which plays a crucial role in battery performance [18]. In such systems, the interface dominates the overall electrochemical behavior due to the higher surface area–volume ratio. Understanding how Li-ions interact with the NMC111 cathode at the interface in low-mass configurations is essential for improving charge transfer kinetics, minimizing resistance, and enhancing interface stability in both liquid and solid-state batteries. In low-mass-loading systems, charge storage mechanisms shift towards surface-dominated processes, such as pseudocapacitance [19,20,21]. Studying these mechanisms is crucial because surface reactions tend to be faster, contributing to high rate capabilities and cycling stability. However, the limited material in low-mass-loading configurations restricts bulk intercalation, so the relative contribution of surface reactions versus bulk intercalation must be well understood to optimize energy storage.

Li-ion transport dynamics are significantly influenced by electrode mass loading. Low mass loading reduces Li-ion diffusion distances, which can improve kinetics and enable faster charge/discharge cycles [22,23]. However, investigating how these improvements in diffusion affect the overall charge storage capacity and energy efficiency is critical for optimizing performance. With lower mass, the relationship between Li-ion diffusivity and capacity retention becomes more complex and needs to be clarified to maximize performance in practical applications.

A key issue in low-mass-loading systems is how much of the active material is electrochemically utilized. The limited amount of active material means that any inefficiency in Li-ion utilization or the loss of charge storage capacity due to side reactions or degradation at the interface becomes magnified. Understanding the factors that influence electrochemical utilization efficiency in low-mass Li-NMC111 systems is fundamental for improving the overall energy density and cycling performance.

Li-metal batteries are known for their high theoretical energy density, which makes them promising for next-generation energy storage [24,25,26]. By pairing Li-metal with the low mass loading of NMC111 and a solid electrolyte, researchers can evaluate the efficiency of the system in terms of maximizing the energy output while minimizing material use. Optimizing the mass loading of NMC111 allows for balancing energy density and cycling stability. Lower mass loading can lead to higher specific capacities per gram of active material, improving the overall energy efficiency.

Li-metal anodes paired with low-mass NMC111 can lead to higher Coulombic efficiency, particularly in solid-state systems. This is critical for reducing capacity fade over long-term cycling. The low mass loading of NMC111 may also enhance fast charging capabilities by reducing the diffusion path lengths for Li-ions, leading to improved rate performance in conjunction with a solid electrolyte that supports stable, high-rate ion transport. By focusing on low mass loading, researchers can potentially reduce the amount of expensive active material (NMC111) needed, which is beneficial for the cost-effectiveness of battery production. This makes it easier to scale up the manufacturing process for commercial applications. Understanding how solid electrolytes perform with lower amounts of NMC111 can inform strategies to maximize performance while minimizing material costs, which is critical for making solid-state Li-metal batteries commercially viable.

Solid-state batteries face several key challenges, including limited ion transport at the electrode/electrolyte interface, higher resistance, and mechanical stability [27,28,29,30]. Researching low-mass NMC111 configurations allows scientists to explore how to overcome these barriers, potentially leading to innovations in solid-state battery design. The combination of low-mass NMC111 with Li-metal and a solid electrolyte could provide new insights into how to optimize battery architecture for improved ion conductivity, longer cycle life, and better mechanical compatibility.

In this manuscript, we present a comparative analysis of Li-ion diffusion and charge storage mechanisms in a low-mass-loaded NMC111 all-solid-state battery (ASSB) and a liquid electrolyte system with comparable NMC111 mass. By evaluating the *b*-values and Li-ion diffusivity during charge and discharge processes, we provide insights into the differing electrochemical behaviors between the solid-state and liquid electrolyte configurations.

## 2. Materials and Methods

### 2.1. Fabrication of a Low-Mass-Loading NMC111 ASSB

NMC111 powder (sourced from TOB, China) was dry ball milled at 400 rpm for 6 h to achieve fine particle size and homogeneity. The battery slurry was prepared with NMC111 as the active material, carbon black (CB), polyvinylidene fluoride (PVDF), and N-methyl-2-pyrrolidone (NMP) in a mass ratio of 8:1:1:30. Initially, PVDF and NMP were stirred for 1 h to form a homogeneous binder solution, after which the NMC111 and CB powders were added. The mixture was thoroughly ground to ensure the uniform dispersion of the active material and conductive additive.

The slurry was uniformly applied onto a 10 μm Al foil substrate with a diameter of 15 mm using a precision applicator to achieve a consistent and even coating. The coated electrode was then dried overnight at 80 °C under vacuum to remove residual solvent and ensure the proper adhesion of the active material to the Al foil. For the thin NMC111 electrode, the mass loading of the active material was carefully controlled to achieve a uniform mass density of 0.57 mg/cm^2^.

### 2.2. Fabrication of LATP Solid Electrolyte Pellet

The LATP powder was ground to eliminate agglomerates and produce fine, homogeneous particles. After calcination, the LATP powder was sieved to achieve a uniform particle size distribution. The sieved powder was then loaded into a cold isostatic press (CIP) for pellet formation, where it was compressed under a pressure of 30 MPa for 30 min. The resulting LATP pellet underwent sintering at temperatures ranging from 1000 °C for 5 h in an argon atmosphere to densify the material. Post-sintering, the pellet was polished to create smooth and flat surfaces, forming a 150 μm thick pellet to enhance electrode/electrolyte contact for optimal performance in electrochemical cells.

### 2.3. Electrochemical and Structural Characterizations

The electrochemical performance of slurry-deposited NMC111 ASSBs was evaluated using CR2032 coin cells, where Li-metal served as both the reference and counter electrodes, and the NMC111-coated electrode acted as the working electrode. A 150 μm thick LATP pellet was employed as the solid electrolyte. For comparative analysis, a liquid electrolyte consisting of 1 M LiPF_6_ (Sigma Aldrich, St. Louis, MO, USA) dissolved in a 1:1 volume ratio mixture of ethylene carbonate (EC) and diethyl carbonate (DEC) was used in an NMC111 Li-metal battery. After assembly, the coin cells were equilibrated for 24 h in an argon-filled glove box to ensure complete electrolyte infiltration and the stabilization of the electrodes.

The electrochemical performance was tested through galvanostatic charge/discharge cycling within a voltage range of 3.0–4.3 V vs. Li/Li^+^ using a battery-testing system (BTS, Neware). The current rates ranged from 1 C to 10 C during cycling. Additionally, cyclic voltammetry (CV) was performed at the scan rates of 0.1, 0.3, 0.5, 0.8, and 1.0 mV/s within the same voltage range to assess the redox behavior of the electrodes.

To support the electrochemical results, the structural characterization of the NMC111-coated electrodes was carried out. X-ray diffraction (XRD) was performed on the freshly fabricated electrodes to verify phase purity and crystallographic orientation. Moreover, electrochemical impedance spectroscopy (EIS) was utilized to evaluate the ionic conductivity of the LATP electrolyte.

## 3. Results and Discussion

**Figure 1:**
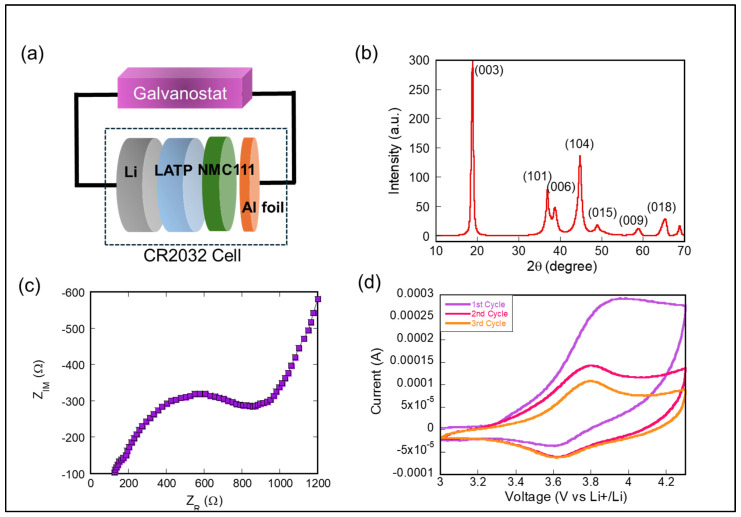
(**a**) Schematic representation of a Li-metal NMC111 ASSB CR2032 cell. (**b**) The X-ray diffraction (XRD) pattern of the NMC111 electrode coated on Al foil. (**c**) The Nyquist plot of a Li/LATP/Li cell configuration. (**d**) The cyclic voltammetry (CV) curves recorded at a scan rate of 0.1 mV·s^−1^.

Figure 1a illustrates the schematic configuration of the Li/LATP/NMC111 ASSB, where LATP is positioned between the Li anode and the NMC111 cathode within a CR2032 coin cell. Figure 1b presents the XRD pattern of the NMC111 electrode material with a low mass loading of 0.57 mg/cm^2^. The distinct diffraction peaks observed confirm the crystalline structure of the NMC111 material, with the sharp peaks indicating the formation of a well-ordered phase. The crystallite size of NMC111, as determined by the Debye–Scherrer method, was measured to be 20 nm. Figure 1c displays the Nyquist plot from EIS, used to assess the ionic conductivity of the LATP solid electrolyte. The calculated ionic conductivity was 2.0 × 10^−5^ S/cm, consistent with the values reported in previous studies, confirming the reliability of LATP as a high-performance solid electrolyte for Li-ion batteries.

We assessed the electrochemical performance of the NMC111 electrode, utilizing Li-metal as both the counter and reference electrodes. CV was performed at a scan rate of 0.1 mV/s within a potential window of 3.0 to 4.3 V vs. Li/Li^+^. Figure 1d presents the CV profiles, revealing a distinct pair of sharp anodic and cathodic peaks corresponding to the Li-ion deintercalation and intercalation processes in the NMC111 structure. The redox peaks, appearing at 3.8 V for oxidation and 3.6 V for reduction, are associated with the Ni^2+^/Ni^4+^ redox couple, confirming stable Li-ion cycling within the NMC111 lattice. The progressive shift of the peaks over the initial cycles suggests an activation process of the electrode material, leading to enhanced stability in the subsequent cycles.

**Figure 2:**
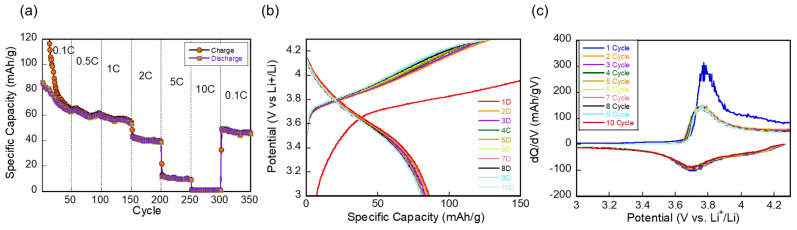
(**a**) Rate capability of the Li-metal NMC111 ASSB at varying C-rates. (**b**) Charge/discharge potential profiles. (**c**) Differential capacity (dQ/dV) versus voltage plots over multiple cycles.

The rate capability of the NMC111-LATP ASSB was investigated by varying the C-rate during the charge/discharge cycles. As shown in Figure 2a, the capacity exhibits a sharp decline at 0.1 C, followed by stabilization at 0.5 C. With increasing C-rates up to 2 C, the capacity gradually decreases, while at higher rates (5 C and 10 C), a pronounced drop in capacity is observed. Notably, upon returning to a lower C-rate (0.1 C) after high-rate cycling, the capacity remains significantly lower than the initial cycle, indicating irreversible capacity loss after high-rate stress.

The potential profile in Figure 2b does not display the distinct voltage plateaus typically associated with phase transitions during Li-ion intercalation and deintercalation. In materials like NMC111, these plateaus arise from two-phase coexistence during ion insertion and removal. However, in the low-mass-loading NMC111 ASSB configuration, this two-phase behavior appears less pronounced due to the limited active material volume. As a result, the voltage changes more smoothly, in contrast to bulk electrodes with higher mass loading, where two-phase transitions are more dominant and lead to more distinct voltage plateaus.

Figure 2c displays the differential capacity versus voltage plot over multiple cycles, where the peaks correspond to the voltages at which phase transitions and redox reactions occur during Li-ion insertion and extraction. The stable peak positions and intensities after the first cycle suggest the high reversibility and robustness of the redox reactions in the low-mass-loaded NMC111 ASSB. Despite the irreversible capacity loss observed in Figure 2a, the close alignment of the curves after the initial cycle highlights the retention of electrochemical activity and continued stability in the redox processes over extended cycling.

**Figure 3:**
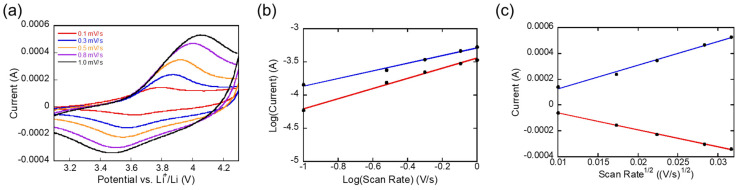
(**a**) CV curves of the Li-metal NMC111 ASSB at varying scan rates. (**b**) The logarithmic plot of current versus scan rate used for *b*-value determination. (**c**) Peak current plotted as a function of the square root of the scan rate to calculate the Li-ion diffusion coefficient. The calculated *b*-values were 0.76 (anodic) and 0.58 (cathodic), and the Li-ion diffusion coefficient was determined to be 1.5 × 10^−9^ cm^2^⋅s^−1^ (anodic) and 7.6 × 10^−10^ cm^2^⋅s^−1^ (cathodic).

CV measurements at various scan rates were conducted to differentiate between the diffusion-controlled and capacitive charge storage mechanisms, as shown in Figure 3a. The electrochemical performance and rate capability of the low-mass-loading NMC111 ASSB are determined by the interplay between diffusion-limited and surface capacitive processes. Diffusion-controlled charge storage, driven by the Li-ion migration through the electrode material, often serves as the rate-limiting factor. In contrast, surface capacitive mechanisms, encompassing both faradaic (surface redox reactions) and non-faradaic (electric double-layer formation) processes, operate more rapidly and contribute to enhanced charge capacity, especially at higher scan rates.

To clarify the contributions of diffusion-controlled, pseudocapacitive faradaic, and non-faradaic charge storage, the sweep rate-dependent CV data were analyzed using the power law equation *I* = *aν^b^*, where *i* represents the current, *ν* is the scan rate, and *a* and *b* are adjustable parameters. The *b*-values, derived from the *log i* versus *log ν* plot in Figure 3b, provide insight into the dominant charge storage mechanisms. A *b*-value of 0.5 indicates diffusion-limited processes, while a *b*-value of 1.0 suggests surface-controlled capacitive behavior. The low-mass-loading NMC111 ASSB exhibits the *b*-values of 0.76 for oxidation and 0.58 for reduction, indicating that both pseudocapacitive and diffusion-controlled mechanisms are active during the charge (oxidation) and discharge (reduction) processes, with a greater pseudocapacitive contribution during oxidation. In the oxidation process, Li-ion deintercalation from the NMC structure occurs more rapidly, likely involving surface or near-surface reactions where the ions interact with redox-active sites. This surface-limited mechanism allows for faster kinetics and higher *b*-values. During the reduction process, Li-ions intercalate into the NMC structure, and this bulk diffusion is slower compared to surface reactions, resulting in a lower *b*-value. The ion transport through the bulk material is more restricted, which slows down the overall reaction kinetics. The difference in the *b*-values between the oxidation (0.76) and reduction (0.58) reactions reflects the asymmetric nature of the charge storage mechanisms. This asymmetry is common in intercalation materials and reflects the inherent differences in ion movement during insertion and extraction processes.

The diffusion coefficient of Li-ions in the low-mass-loading NMC111 ASSB was determined using the CV data across different scan rates applying the Randles–Sevcik equation:

$$I_{p}=0.4463nFAC_{Li}({\frac{nF\nu D_{Li}}{RT})}^{\frac{1}{2}}$$

where *I_p_* is the peak current, *n* the number of electrons transferred, *F* the Faraday constant (96,485 C∙mol^−1^), *C_Li_* the initial concentration of Li^+^ ions, A the electrode surface area, *D_Li_* the diffusion coefficient, *R* the gas constant, and *T* the temperature. By plotting *I_p_* against *ν*^1/2^, as shown in Figure 3c, the diffusion coefficient was estimated to be 1.5 × 10^−9^ cm^2^⋅s^−1^ (oxidation) and 7.6 × 10^−10^ cm^2^⋅s^−1^ (reduction), indicating efficient Li-ion transport in the low-mass-loading NMC111 ASSB.

**Figure 4:**
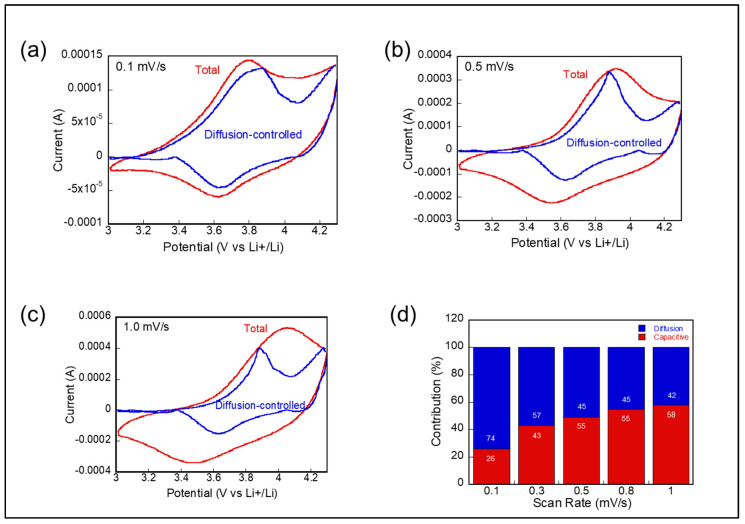
(**a**–**c**) Comparative analysis of diffusion-controlled and surface capacitive contributions at varying scan rates. (**d**) Contribution of charge storage mechanisms at different scan rates, with surface capacitive effects increasing at higher scan rates.

By analyzing the CV sweep rate dependence, we distinguished the capacitive and diffusion-controlled contributions to the current, as illustrated in Figure 4. The equation *i*(*V*) = *k*_1_*ν* + *k*_2_*ν*^1⁄2^ was used to describe the current at a fixed potential, where *k*_1_*ν* represents the contribution from surface capacitive effects, and *k*_2_*ν*^1/2^ corresponds to diffusion-controlled Li-ion insertion. To further quantify these contributions, the equation was rearranged to *i*(*V*)⁄*ν*^1⁄2^ = *k*_1_*ν*^1⁄2^ + *k*_2_, allowing for the separation of the capacitive and diffusion-controlled processes. By plotting the current against the sweep rate, *k*_1_ and *k*_2_ were extracted from the slope and intercept of the linear plot at each potential.

The comparison between the diffusion-controlled contribution (blue line) and the total charge in Figure 4 shows an increasing dominance of capacitive behavior at higher scan rates. As the scan rate increased from 0.1 to 1.0 mV/s, the ratio of the total charge to the capacitive charge was calculated based on the area under the curve in Figure 4a–c. The results indicate that the contribution of surface capacitive effects significantly rises with increasing scan rate, highlighting their growing influence on the overall charge storage at higher rates, as shown in Figure 4d.

We compared the rate capability, charge storage mechanisms, and Li-ion diffusivity between the low-mass-loading NMC111 ASSB and a liquid electrolyte system with a comparable NMC111 loading mass density, as summarized in Table 1. When investigating the low-mass-loading NMC111, which is ideal for probing the electrode/electrolyte interface in both liquid and solid electrolyte cases, a large difference in Li-ion diffusivity was observed. Notably, the Li-ion diffusion coefficient in the liquid electrolyte system was significantly higher than that in the solid electrolyte. Additionally, in the low-mass-loading NMC111 ASSB, the *b*-values varied between the anodic and cathodic reactions, whereas in the liquid electrolyte system, the *b*-values were relatively consistent for both processes. This variation in *b*-values in the ASSB can be attributed to differences in ionic transport and the formation of space charge layers at the electrode/electrolyte interface, which are less pronounced in liquid electrolyte systems due to superior ionic mobility and interfacial contact [31,32,33].

In liquid electrolyte systems, the Li-ions have high mobility due to the fluid nature of the electrolyte, resulting in fast and symmetric ion transport during both the anodic (oxidation) and cathodic (reduction) processes. This typically leads to similar *b*-values for both reactions, as the rate-limiting factors for Li-ion movement are comparable during insertion (reduction) and extraction (oxidation). In contrast, solid electrolytes, such as LATP, exhibit lower Li-ion mobility due to their rigid structure and often higher interfacial resistance at the solid–solid contact points between the electrolyte and electrode materials. More critically, the migration of Li-ions at the electrolyte/electrode interface, driven by the electrochemical potential difference, induces the formation of a space charge layer, creating an internal electric field [32,33]. This field shifts the original energy levels, causing band bending and forming energy barriers that hinder charge carrier transfer. These barriers can obstruct the movement of electrons and holes, influencing the oxidation/reduction reactions at the electrolyte. As a result, asymmetric charge storage mechanisms may arise, along with alterations in the charge transfer efficiency at the interface, ultimately affecting the overall battery performance.

This reduced ionic transport in solid-state systems can lead to asymmetry in the electrochemical behavior during charge and discharge, particularly at higher rates. In liquid electrolyte systems, the Li-ions have high mobility due to the fluid nature of the electrolyte, resulting in fast and symmetric ion transport during both the anodic (oxidation) and cathodic (reduction) processes. This typically leads to similar *b*-values for both reactions, as the rate-limiting factors for Li-ion movement are comparable during insertion (reduction) and extraction (oxidation). In contrast, solid electrolytes, such as LATP, exhibit lower Li-ion diffusivity due to their rigid structure and often higher interfacial resistance at the solid–solid contact points between the electrolyte and electrode materials. This reduced ionic transport in solid-state systems can lead to asymmetry in the electrochemical behavior during charge and discharge, particularly at higher rates.

The lower diffusion coefficient in NMC111 ASSB exacerbates the rate-limiting effects of Li-ion transport, particularly during the reduction (cathodic) process, when Li-ions must diffuse into the bulk of the NMC111 electrode. This slower diffusion results in a *b*-value closer to 0.5 for the cathodic reaction, reflecting a more diffusion-controlled process. During the oxidation (anodic) process, Li-ions are deintercalated from the surface of the NMC111, where pseudocapacitive (surface-driven) reactions may dominate, resulting in a higher *b*-value (closer to one). The faster kinetics during oxidation, driven by surface interactions rather than bulk diffusion, lead to the observed difference in the *b*-values between the anodic and cathodic processes in the solid-state system.

Solid electrolytes often suffer from higher interfacial resistance at the electrode/electrolyte interface, further slowing down Li-ion transport, especially during the intercalation (cathodic) process. This resistance can hinder the smooth flow of ions into the electrode material, contributing to the lower *b*-value during reduction, as ion transport becomes more limited by diffusion. Liquid electrolytes, on the other hand, maintain excellent wetting of the electrode surfaces, reducing interfacial resistance and allowing for more uniform Li-ion transport in both directions (insertion and extraction), leading to more similar *b*-values for both anodic and cathodic reactions.

## 4. Conclusions

This study investigated the electrochemical performance of a low-mass-loading NMC111 ASSB using LATP as a solid electrolyte compared to a low-mass-loading Li-NMC111 battery with a liquid electrolyte. Both systems exhibited a similar *b*-value of 0.7 during oxidation (anodic) reactions, indicating predominant pseudocapacitive behavior, suggesting that the surface reaction-dominated charge storage mechanisms in the solid-state battery are comparable to those in the liquid electrolyte system. However, the Li-ion diffusion coefficient in the liquid electrolyte was significantly higher, contributing to its superior rate capability.

The difference in the *b*-values between the anodic (oxidation) and cathodic (reduction) reactions in the low-mass-loading NMC111 ASSB, compared to the similar *b*-values in the liquid electrolyte systems, is primarily due to the lower diffusion coefficient and higher interfacial resistance in solid electrolytes. These factors result in a more diffusion-limited process during reduction, while oxidation remains surface-driven and faster. In liquid electrolytes, the higher ionic mobility and better electrode/electrolyte contact lead to more symmetric behavior in both the charge and discharge processes, with similar *b*-values for the anodic and cathodic reactions. The formation of space charge layers and high interfacial resistance at the LATP/NMC111 interface are critical factors leading to asymmetric diffusivity and hindered performance in ASSBs. These phenomena primarily affect the Li-ion transport during intercalation, resulting in slower kinetics and lower overall battery performance. Overall, this work demonstrates that solid-state Li-NMC111 batteries are a promising alternative for safer energy storage but require further optimization in ionic conductivity to match the high-rate performance of liquid electrolyte systems. Further, while low-mass-loading configurations may not immediately provide the high energy densities required for mainstream commercial applications, they offer significant potential in specialized systems such as fast-charging batteries, thin-film technologies, and hybrid architectures. By leveraging their advantages—such as enhanced interface stability, fast ion transport, and reduced material usage—these designs can find practical application in high-power, lightweight, and energy-efficient commercial battery systems.

## Figures and Tables

**Table 1:** Comparative summary of low-mass-loading NMC111 batteries with solid and liquid electrolytes.

		*b*-Value	Diffusion Coefficient (cm^2^⋅s^−1^)	Fraction of Capacitive to Diffusive Charge Storage (1.0 mV/s)	Average Specific Capacity (mAh⋅g^−1^) (0.5 C)	Average Specific Capacity (mAh⋅g^−1^) (2 C)	Average Specific Capacity (mAh⋅g^−1^) (5 C)
With Liquid Electrolyte	Anodic	0.70	1.64 × 10^−8^	1.78	61	40	11
	Cathodic	0.77					
With Solid Electrolyte	Anodic	0.76	1.50 × 10^−9^	1.38	90	54	28
	Cathodic	0.58					

## Data Availability

The raw data supporting the conclusions of this article will be made available by the authors on request.
